# A critical appraisal and questions on the immunological evolution of mogamulizumab-induced drug eruption into cutaneous adult T-cell leukemia/lymphoma infiltration

**DOI:** 10.1016/j.jdcr.2025.08.041

**Published:** 2025-10-07

**Authors:** Parth Aphale, Himanshu Shekhar, Shashank Dokania

**Affiliations:** Research and Development Cell, Dr. D.Y. Patil Vidyapeeth (Deemed to be University), Pimpri, Pune, Maharashtra, India

**Keywords:** adult T-cell leukemia/lymphoma (ATLL), CD4+ T-cell-dominant infiltrate, mogamulizumab-induced

*To the Editor:* We read with great interest the recent case report by Inayoshi et al detailing the immunological evolution of a mogamulizumab-induced drug eruption into adult T-cell leukemia/lymphoma cutaneous infiltration. This article offers a valuable, longitudinal perspective on a critical diagnostic challenge in managing adult T-cell leukemia/lymphoma patients. The use of serial biopsies and double immunofluorescence staining to demonstrate a clear shift from CD8+ T-cell predominance to a CD4+ T-cell-dominant infiltrate provides a compelling and insightful framework for clinical differentiation.^1^

While the authors’ findings are a significant contribution, they also raise several questions regarding the complexity of the case. The patient was initially treated with both mogamulizumab and CHOP chemotherapy. While the authors state that CHOP was not associated with drug eruptions, the combined administration of these therapies may have complicated the initial immunological landscape and could have influenced the observed immune cell dynamics. Could the synergistic or antagonistic effects of CHOP on the T-cell subsets have played a role in the initial CD8+ T-cell response? Furthermore, the reported shift from a reactive to a neoplastic infiltrate raises a fundamental question about the underlying mechanism. Is this a true evolution of the same T-cell clone, or is it a process of competitive exclusion, where a reactive CD8+ T-cell infiltrate is gradually overwhelmed and replaced by the expanding, neoplastic CD4+ adult T-cell leukemia/lymphoma clone? Investigating the T-cell receptor clonality of both the early and late infiltrates could provide a definitive answer to this.^2^^,^^3^

Moreover, while the use of repeated biopsies is a robust method, it is invasive and may not be a practical long-term monitoring strategy. We must ask if less invasive biomarkers could signal such a critical transition. Recent research has explored the use of serum biomarkers and molecular profiling of skin samples to distinguish inflammatory from neoplastic processes in cutaneous T-cell lymphomas. For instance, studies have identified specific serum cytokines and circulating tumor DNA as potential noninvasive tools for monitoring disease progression and treatment response. Could the shift in CD4/CD8 ratio observed in the skin also be reflected in the peripheral blood, providing a more accessible marker for this transition^4^^,^^5^?

In conclusion, this case report skillfully highlights a crucial diagnostic and pathological dilemma. While it provides a strong foundation for using immunophenotypic shifts as a diagnostic marker, it also paves the way for further research into the molecular mechanisms of these transitions and the development of less invasive monitoring strategies.

## Conflicts of interest

None disclosed.
